# Supplementary material for: Are carbon emissions trading and green financial instruments synergistic? -Comprehensive quantitative research based on content analysis
Source: PLoS One. 2024 Mar 7;19(3):e0298601. doi: 10.1371/journal.pone.0298601 (PMC10919601; doi:10.1371/journal.pone.0298601)
Supplement: S1 Data — (ZIP) [file pone.0298601.s001.zip › Data/地级市Z_F工作报告.zip!/╝¬┴╓/═¿╗»╩╨/└┤╫╘╣1⁄2╓┌║┼í╛┬φ┐╦╩2╛▌═°í┐_2013.docx]

一、过去五年和2012年工作回顾

　　2008年以来的五年，是通化经历重大挑战、取得重大成就的五年。在市委的坚强领导下，在市人大、市政协的监督支持下，市政府带领全市人民，有效应对国际金融危机影响和特大洪涝灾害等考验，抓项目、兴产业、惠民生、促发展，圆满完成了市六届人大历次会议确定的目标任务。

　　——经济持续快速发展。预计：2012年实现地区生产总值910亿元，是2007年的2.6倍，年均增长16.3%；全口径财政收入104.9亿元，是2007年的2.9倍，年均增长24.1%。地方级财政收入62.6亿元，是2007年的3.9倍，年均增长31.1%；规模以上工业总产值1360亿元，是2007年的3.2倍，年均增长25.9%；社会消费品零售总额335.8亿元，是2007年的2.4倍，年均增长19.2%。主要指标超额实现翻番目标，多项指标增速一直位居全省前列。省下达的节能减排约束性指标全面完成。

　　——老工业基地振兴成效显著。五年完成固定资产投资3172.5亿元，是上个五年的4.5倍；实施3000万元以上项目3045项、亿元以上项目628项，3000公斤人胰岛素冻干粉和7000万支针剂、50万吨特优级食用酒精、20万吨啤酒、500千伏输变电等462个超亿元项目建成投产；1014个项目纳入国家投资计划，争取资金61.3亿元。三次产业比例调整到9.8：52.6：37.6。医药、冶金、食品三大支柱产业产值分别达到547亿元、194亿元、178亿元，是2007年的4.4倍、1.3倍、4.7倍。新口径规模以上工业企业发展到520户，国家高新技术企业发展到23户，新创中国驰名商标7件、“中华老字号”品牌3件、国家地理标志保护产品16种。省级以上农业产业化龙头企业发展到57户，农产品加工量突破215万吨。畜牧业和园艺特产业产值年均分别增长21%、17.5%。完成人参基因组图谱制作，16户企业列入全省人参药食同源试点，人参产业产值年均增长29%。欧亚购物中心、义乌国际商贸城和中东城市广场等商贸项目建成运营。实施旅游项目120项，旅游收入年均增长32%。市区规模以上工业总量和地方级财政收入分别是2007年的2.5倍和2.7倍。县域全口径财政收入增长3.1倍，梅河口市达到26亿元，集安市、通化县、柳河县、辉南县和东昌区均过10亿元。二道江区列入全国第三批资源枯竭城市转型试点、首批矿山地质环境综合治理试点。

　　——城乡面貌发生巨大变化。城市总体规划通过省政府批准，5个专项规划编制完成。市区投入资金167亿元，完成50多项重点工程，城市功能和品位显著提升。“暖房子”工程改造面积732万平方米，基本实现老旧楼房“整体翻新”；拆迁棚户区273万平方米，开工建筑面积415万平方米；完成二道河改造、滨江东路北段、三期平湖、供热三期、跨江人行景观桥等工程，建设改造115条街巷路和沿江景观带、玉皇山、倚江园等5个公园；滨江沿河、城区街路绿化美化亮化水平大幅提高，人均绿地面积达到11.9平方米，路灯达到8.4万盏；江南新区初具规模，配套服务功能日趋完善；城区周边17.8万人全天用水和3.6万户居民用气问题得到解决，集中供热规模达到1280万平方米；医疗废物处理中心建成使用；空气环境质量优良天数由301天升至335天。市区和县城污水处理厂、垃圾无害化处理厂全面建成，饮用水源水质全部达到国家标准。完成新农村建设项目1.8万项，全市行政村达到“四清四改两化”标准。完成水利工程256项，修建主要江河堤防65.2公里。植树造林91.3万亩，绿化村屯1303个，哈泥河自然保护区晋升国家级保护区，连续58年无重大森林火灾。

　　——通道工程建设实现历史性突破。建设公路3215公里，公路总里程达到6415公里，其中高速公路215公里，普通公路6200公里。通沈、通丹高速公路全线通车，梅沈、营松高速公路通化段建成通车，通丹铁路竣工，进关出海快捷通道全面打通，通化区位显著提升。

　　——开发开放和体制机制创新步伐加快。医药高新区与开发区实现整合，申报国家级高新区进展顺利。县域开发区全部晋升省级开发区。通丹经济带列入省“十二五”规划。全市引进资金3246亿元、亿元以上项目1262项，实际利用外资9.7亿美元，美国辉瑞、韩国浦项两户世界500强和四环、步长等一批国内知名企业落户通化。通钢与首钢实施重组。在全省率先完成集体林权制度改革。粮食流通、农业保险等农村改革取得成效。组建国土资源交易中心，土地出让实现规范化、制度化。招拍挂出让土地1391公顷，新增耕地7762公顷。民营经济主营业务收入年均增长34.6%，新增缴税超千万元企业60户。通天酒业、双龙化工、益盛药业成功上市，金宝药业、万家福食品、远通路桥在天津股交所挂牌融资。吉林银行通化分行和招商银行通化分行建成开业，小额贷款公司发展到49家。

　　——社会各项事业蓬勃发展。科技创新“310”工程顺利完成，列入国家和省科技专项184项，高新技术产业增加值年均增速超过20%。教育事业全面发展，新建和改造校舍66.5万平方米，校舍安全工程全面完成，通化技师学院正式组建，市青少年宫投入使用。公共文化服务体系逐步完善，阶段性文化体制改革基本完成，文化产业整体实力不断提升。艺术创作获国家和省级奖项66项，组织广场演出、送戏下乡2300余场，非物质文化遗产列入国家级名录3项、省级名录34项，举办了两届松花砚（石）文化节系列活动，关东文化产品交易市场被确定为吉林省文化产业示范园区。新型农村合作医疗参合率达到99.96%，基本药物制度全面推开，新建扩建医疗卫生机构16个，各类传染病得到有效控制。群众体育活动丰富多彩，竞技体育获省级以上金牌433枚。全市99个社区标准化办公用房建设全部完成，社区“五有一创”工程成果显著。社会事业基础设施得到完善。广播电视、人口计生、妇女儿童、老龄、残疾人事业全面发展，民族、宗教、外事、侨务、审计、统计、人防、气象、地方志、地震监测、红十字会、慈善和军民共建等工作都取得了新成绩。

　　——人民生活进一步改善。制定实施民生工作计划，每年为群众办100余件实事。城镇居民人均可支配收入达到21550元，比2007年增加9262元，年均增长11.9%；农民人均纯收入达到9100元，比2007年增加4754元，年均增长15.9%。城镇新增就业28.4万人，新增社会保险参保69万人。企业退休人员养老金提高65.4%，养老、工伤、生育保险和住房公积金参保人数分别增长12.5%、70%、80.5%、29.3%，城乡居民养老保险和医疗保障实现全覆盖。城市低保月标准和农村低保年标准分别增长1.1倍和1.8倍。年均救助困难群众16万人。“八路安居”工程改造建设面积1097.7万平方米，改善了17.7万户城乡低收入群体住房条件。建成农村饮水安全工程1289处，79.9万农村人口受益。

　　——民主法制建设和社会管理服务水平不断提高。办理人大代表建议832件、政协提案781件。“五五”普法全面完成，“六五”普法启动实施。政务大厅投入运行，行政审批制度改革继续深化。政府法制工作得到加强。“N+1”调解矛盾纠纷工作模式在全国推广。加大信访工作创新力度，“三级三会”解决信访难题、终结信访案件成效显著。深入推进平安通化建设，全市政治稳定、社会稳定、治安稳定、边境稳定，人民群众安全感测评满意率逐年提升。精心组织安全生产“五项工作”，加强生产、消防、道路交通、食品药品等重点领域和行业安全隐患排查整治，保障公共安全的长效监管体制进一步健全。市长公开电话受理群众来电24.5万件。深化政行风民主评议和纠风专项治理，软环境进一步改善，反腐倡廉取得新成效。认真开展主题教育和创先争优活动，各级公务员服务基层、服务群众的积极性明显增强。省委、省政府先后在通化召开16次现场会和经验交流会，获得全省绩效考核评比优秀档次。

　　2012年，全市上下攻坚克难，推动经济社会实现平稳较快发展。与上年相比，地区生产总值增长15%，全口径财政收入增长25.6%，地方级财政收入增长30.1%，固定资产投资增长32%。

　　一是强力推进项目建设和招商引资。续建和新建项目数量分别增长30%和35%，大项目数量、质量和投资额度创历史最好水平。四长医药工业园、人胰岛素注射剂三期工程、通化生物科技园等280个超亿元项目开工建设，5万吨镁合金、浦项钢材加工中心、修正斯达舒扩能改造、康美人参产业园、凤形耐磨等一批重点项目建成投产。通化机场建设工程基本竣工，现已具备开航条件。通梅高速公路开工，官道岭互通立交桥主体工程完工，通靖高速公路通过国家发改委批复，通化列入国家“十二五”公路运输主枢纽城市。招商引资到位资金938亿元，引进亿元以上项目340项。三一重工、中国铁建、中钢研、晋商联盟等知名企业进入通化。

　　二是促进工业增长和产业跃升。实体经济发展提速，规模以上工业增加值增长12%，利润达到56亿元，净增新口径规模以上工业企业87户。中国中医药展览交易基地落户通化，首届中国中医药产业博览会成功举办，9户医药企业完成新版GMP改造。通钢装备升级改造进展顺利，60万吨精品棒材、60孔焦炉即将投产。有效化解通葡股份退市风险，成功摘掉“*ST”并完成企业重组；鸭绿江河谷葡萄产区规划建设启动，葡萄酒产业产值增长30%。出台加快培育发展战略性新兴产业、提升特色生态资源产业实施意见。红星美凯龙家居广场开工建设，长白山医药物流中心建成运营，欧亚购物中心扩建工程完工。开通集安至松江河旅游专线，实施振国养生谷、高句丽王家园林等旅游项目，五女峰晋升4A级景区，四季风光松花套砚获全国旅游商品大赛金奖，接待国内外游客687万人，实现旅游收入68.9亿元。“精彩通化”上线运行，“智慧通化”签约。通化信用担保公司和双基证券咨询公司挂牌运营。

　　三是加大城市建设和管理力度。市区改造棚户区61.5万平方米。“暖房子”工程完成改造370万平方米，是省下计划的4.1倍；同步改造陈旧供热管网200公里，撤并改造小锅炉房和换热站36座；完成部分旧住宅小区地下排水设施和架空线路落地改造。北出口改造全面竣工，江南新区路网升级改造、绿化美化亮化等工程如期完成。解决了东岭、西山等区域1.8万户居民全天用水问题，新增煤气用户6000户，亮化黑楼道300栋。饮用水源保护、烟气脱硫、污染治理等工程按时完工。国有土地上房屋征收依法有序高效推进，全市完成征收面积103万平方米。全国首个国土资源政策法律实验基地在通化揭牌。通化保障性安居工程代表吉林省接受国家住建部检查。被国家批准利用公积金贷款支持保障性住房建设试点城市。城市管理、交通管理、物业管理得到加强，城市环境综合整治和铁路沿线环境治理成效明显，实现了省级园林城市创建目标。

　　四是加快发展农业农村和县域经济。粮食产量达到44亿斤，创历史新高。现代畜牧业四年提升计划启动实施，建成标准化牧业小区140个。新增棚膜蔬菜5400亩，现代农业产业园龙头无公害棚膜基地投入使用。实施千万元以上农业产业化项目20项，吉煤特色农牧产业基地建设进展顺利，中药材、蓝莓等产业基地发展到50万亩，食用菌种植达到500万平方米。新开发认证绿色有机无公害农产品20个。新农村建设深入推进，115个省级示范村全部达标，其余行政村40%达到先进村标准。159个农村环境连片整治示范项目顺利完成，通化县成为全国农村环境连片整治唯一整体推进县。浑江干流整治、水库除险加固等65项水利工程按期完工。省级林业龙头企业发展到26户，回收被侵占林地52万亩。3个县（市）进入全省县域综合发展考评前十位，梅河口市获二等奖，辉南县、柳河县获三等奖，我市连续获组织推动奖。

　　五是推动民生改善和社会进步。年初确定的112件民生实事全面完成。企业退休人员养老金月均增加179元，高于全国平均水平。城市低保月标准提高到315元，农村低保年标准提高到1987元，新型农村合作医疗政府补贴增至240元。“八路安居”工程实现从城镇到农村、矿区、林区、垦区全覆盖。改造农村危房25898户、工矿棚户区27.7万平方米，建成廉租房12.5万平方米，为1.8万户城镇低收入家庭发放了住房租赁补贴。市本级争取上级财政专项资金28.4亿元，比上年增加4.9亿元；偿还地方政府性债务5.2亿元。市科技文化活动中心和城市规划展览馆主体工程完工，中心医院外科大楼、十三中迁址等工程开工建设，残疾人康复中心和二道江体育馆投入使用。“文化年”成果丰硕，组织开展名家名人名品评选和中国文化遗产日等系列活动，32人列入第二批省级非物质文化遗产传承人，在全省率先实现农村文化大院全覆盖，市歌舞剧院完成转企改制，电视剧《远去的飞鹰》荣获金鹰奖等多项国内外大奖。成功举办了第四届少数民族传统体育运动会。

　　六是切实维护社会稳定。创新社会管理，加强信访工作，排查调处矛盾纠纷11200件，集中化解信访积案391件，进京非正常访连续4个月实现零登记，“四无访”登记创全省最好成绩。大力推进社会治安防控“八张网”建设，严厉打击各种违法犯罪，刑事发案率下降15%，命案侦破率100%，社会治安大局持续平稳。深入开展安全生产隐患排查治理，事故起数和死亡人数实现“双下降”。食品安全检验中心投入使用，食用油、食品添加剂、蔬菜农药残留、畜禽屠宰等食品安全专项整治扎实推进。依法依规稳妥处置“问题胶囊”事件，全面加强医药生产企业质量安全监管、检验检测能力和标准化建设，在较短时间内恢复了生产和市场，医药产业在逆境中得到发展。

　　五年来，通化列入国家老工业基地试点城市、知识产权试点城市；被认定为中国医药城、松花砚之乡、人参产业基地、首批国家新型工业化产业示范基地和低碳国土实验区；荣获联合国中国区环境规划优秀示范城市、全国双拥模范城“七连冠”、质量兴市先进市、“双保工程”成效显著城市、科技管理先进单位、中国最具国际影响力旅游城市、最具人文情怀城市、食品安全十佳城市等多项殊荣。在2012中国最具幸福感城市评选中，通化位列全国地级以上城市第22位。

　　各位代表！五年来取得的成绩极为不易，这是市委正确领导的结果，是市人大、市政协监督支持的结果，是全市各族人民团结奋斗的结果，是社会各界共同参与的结果。在此，我代表市政府，向辛勤工作在各行各业的全市人民，向给予政府工作大力支持的人大代表和政协委员，向各民主党派、工商联、无党派人士和人民团体，向驻通国省属单位、驻通部队和武警官兵，向所有关心支持通化发展的同志们、朋友们，表示衷心的感谢并致以崇高的敬意！

　　在肯定成绩的同时，我们也清醒地看到存在的问题，经济总量还不大，产业结构还不尽合理，一些企业发展面临困难，转方式、调结构、增效益任务繁重；城镇化基础薄弱，城市发展受空间制约比较突出；城乡居民收入水平还不高，部分群众生活还很困难；经济与社会发展不够协调，影响社会和谐的因素依然存在，创新社会管理、维护社会稳定需要继续加大力度；部分公职人员思想观念、素质能力和工作作风不完全适应形势任务要求，公共服务能力和社会管理水平有待进一步提高。对这些问题，我们正在采取措施，认真加以解决。

　　二、今后五年发展目标和主要任务

　　今后五年，是通化深化改革开放、加快转变发展方式的攻坚阶段，是科学发展、加快振兴的关键时期。全球新一轮经济调整、科技创新和产业转型，党的十八大提出到2020年实现全面建成小康社会宏伟目标，为我们提供了极为有利的发展条件。但同时，宏观经济不稳定不确定因素依然很多，区域竞争更加激烈。我们必须进一步增强机遇意识、忧患意识和责任意识，励精图治，奋发图强，努力开创通化振兴发展新局面。

　　今后五年，市政府工作的指导思想是：以科学发展观为指导，深入贯彻落实党的十八大和省十次党代会、市六次党代会精神，紧紧围绕全面建成小康社会总目标，统筹推进工业化、信息化、城镇化、农业现代化，全面加强经济建设、政治建设、文化建设、社会建设和生态文明建设，坚定不移抓项目、兴产业、惠民生、促发展，更加注重保持经济平稳较快发展和积蓄发展能量，更加注重优化经济结构和转变发展方式，更加注重深化改革开放和提升自主创新能力，更加注重加快特色城镇化发展和生态环境保护，更加注重改善民生和创新社会管理，加快建设东北东部地区中心城市、吉林省东南部新型工业基地、中国北方宜居山水城市，推动通化科学发展、加快振兴，让全市城乡居民生活得更加美好。

　　做好今后五年工作，必须始终坚持解放思想，用新思维新思路破解难题、谋划发展；必须始终坚持发展第一要务，全力做大经济总量，提高发展质量和效益，努力实现经济社会全面协调可持续发展；必须始终坚持以人为本、服务为先，竭力满足人民日益增长的物质和精神文化需求，不断提升公民道德素养和文明水平，切实提高群众的满意度和幸福感。

　　今后五年的主要奋斗目标是：

　　——综合实力再上新台阶。经济总量在现有基础上实现翻番，占全省比重明显提高，人均GDP超过全省平均水平。2017年，地区生产总值力争达到2000亿元，全口径财政收入和地方级财政收入分别达到240亿元和115亿元，规模以上工业总产值达到3000亿元，社会消费品零售总额达到660亿元。

　　——经济结构得到新优化。三次产业结构力争调整到8：52：40。以支柱产业、优势产业、战略性新兴产业、特色生态资源产业为支撑的现代产业体系在全国形成较强的竞争力、影响力，民营经济占GDP的比重达到80%以上，工业化、信息化、城镇化、农业现代化同步发展格局基本形成。

　　——城乡建设实现新突破。城市规划区面积超过200平方公里，中心城区完成新一轮改造提升，老城区实现“整体翻新”，江南商贸新城和湾湾川生态新城形成规模，实现国家卫生城市、园林城市、环保模范城市和全国文明城市创建目标。城乡一体化进程加速推进，县城和中心镇基础设施更为完善，承载功能明显增强，特色品位更加鲜明。

　　——改革开放迈出新步伐。思想观念、发展模式、体制机制、对外开放、企业管理和城市管理等各领域的创新深入推进，市场化改革进一步加快，科技创新能力显著增强，投资创业环境更加优良，参与国内外竞争的能力显著提高。

　　——人民生活达到新水平。城乡居民收入增速超过全省平均水平，城镇居民人均可支配收入力争五年实现翻番、达到43000元，农民人均纯收入力争五年左右翻番、达到18200元。群众享有充分的就业、完善的社会保障、满意的公共服务和丰富的精神文化生活，民生保障水平进入全省前列。

　　——社会管理取得新成效。建立起与开放、动态、信息化环境相适应的社会管理体系和以人为本的社会管理新模式，健全基层社会管理体制，提高社会安全管理能力和水平，维护社会公平正义、和谐稳定，建设成为全国平安城市。

　　今后五年，突出抓好以下工作：

　　（一）继续实施投资拉动、项目带动和创新驱动。固定资产投资累计完成7000亿元，年均增长20%以上。构建以“一大园区、两大基地、三大工程、四大板块、五大新兴产业”为主的项目支撑体系，做大经济总量，提升综合实力。滚动实施亿元以上项目1000项，其中10亿元以上项目80项，谋划储备超亿元项目1000项，工业投资占比超过60%。集聚创新资源，规划建设10个国家和省级特色产业示范基地、10个重大技术产业化示范项目、10个省级企业科技创新中心，组建一批科技企业孵化器、重点实验室、院士和博士后工作站、科技创新公共服务平台，为把通化建成国家重要的科技研发与创新基地、东北东部发展的区域创新极打下坚实基础。

　　（二）推动产业结构优化升级。实施支柱优势产业跃升、战略性新兴产业培育、特色生态资源产业提升计划，促进工业化与信息化深度融合。规模以上工业企业达到800户，年产值亿元以上企业达到300户、10亿元以上企业达到100户、百亿元以上企业达到4户。建设千亿规模生物产业基地，突出发展现代中药、生物制药，加速医药产业整合重组、存量盘活，研发一批创新药物，全面完成新版GMP改造，打造全国医药强市和国际化程度较高的“医药名城”。建设新型钢铁基地，支持通钢淘汰落后产能、完成装备升级改造，推进冶金产业园区与通钢对接，发展地方兴钢产业，打造“精品钢城”和钢铁深加工产业集群。建设绿色食品和保健品产业基地，加大长白山绿色食品资源、特色生态资源开发转化力度，提高资源加工深度。大力发展新材料、新能源、节能环保、先进装备制造等战略性新兴产业。做大做强金厂镁合金新区。2017年，医药产业产值达到1000亿元，冶金产业产值达到500亿元，食品产业产值达到560亿元，特色生态资源产业占食品产业的比重超过50%，战略性新兴产业产值年均增长30%以上，能源、机械制造、建材、化工等产业的竞争优势明显提升。实施品牌战略和标准化战略，创建全国知名品牌示范区和质量强市示范市。推进现代物流、商贸、文化等服务业提速发展，培育旅游文化商贸复合型支柱产业，把通化建设成为东北亚重要旅游目的地和东北东部物流中心、商贸中心。依托生态环境优势，加快建设中国养老养生休闲度假基地。

　　（三）推进特色城镇化。突出“两轴两区一带”规划建设，形成具有通化特色的城镇发展体系。实施通化都市区率先突破，加快旧城改造、新区开发、新城建设，把主城区打造成为核心区，把二道江、通化县城区打造成为辅城区，把“五鸭铁”、二密、金斗、三源浦组团打造成为拓展区和特色功能承载区。适应城市规模扩张需要，全力推进基础设施重点工程，拉大中心城市发展框架。抓好老旧地下管网改造、架空线路落地和市政设施功能提升，建成“气化通化”、“数字通化”等资源保障工程，实施水利工程382项，新增变电容量70万千伏安、集中供热能力320万平方米，建设应急备用水源，筹建会展中心。实施交通畅通工程，打通丁字路、断头路，有效解决中心城区停车难和交通拥堵问题。完成光复路、福明路等出口改造，实现与绕越线立交连接。健全科学化、精细化城市管理长效机制。建设“通梅经济走廊”、“通梅集城镇发展轴”和“梅柳辉经济协作区”，推动县城扩容和城关镇、中心镇发展，增强承载力、带动力。加强农村水电气路等基础设施建设，整体提升新农村建设水平。2017年，全市城镇化率达到60%。

　　（四）加快农特产业发展崛起。建设现代农业产业体系，形成粮食、畜牧业、园艺特产业、林业、农产品加工多业并举的特色农业发展格局。大力推进农业产业化，强化龙头企业带动作用，促进农业增效、农民增收。实施优质粮食丰产工程，发展设施农业，落实“菜篮子”责任制，提高农业综合生产能力。推动畜牧业产加销一体化发展。推进人参种植规模化、质量标准化、加工精深化、产业集约化，建设人参产业园区和交易中心，实现人参产业在全省率先发展。推进以鸭绿江河谷葡萄产区为重点的山葡萄种植基地建设，建立山葡萄标准体系，积极发展葡萄酒庄，扶持通葡、通天等骨干企业做大做强，把通化葡萄酒打造成东北地区葡萄酒产业的龙头。抓好林下种植养殖业、林特产品加工业和森林食品开发，促进柳河国家级苗木交易市场加快发展。加强农产品品牌培育、科技创新和市场开发，健全农产品质量安全检疫检测体系。2017年，畜牧业、园艺特产业、人参产业、林业产值分别达到220亿元、450亿元、260亿元、400亿元，农产品加工转化能力达到400万吨，农特产业成为新的支柱产业。

　　（五）进一步深化改革、扩大开放。统筹户籍、土地等城镇化综合配套改革，推动城乡社会保障体系衔接。支持企业引进战略投资者，推进跨行业、跨区域合作重组。深化地方金融体制改革，加大域外金融机构引进力度，扩大资本市场融资。抓好行政管理体制、财税体制、资源性产品价格、收入分配制度和国有林场等改革。推动和支持全民创业，民营企业突破万户大关。密切与辽宁沿海经济带和长吉图对接联系，深化与东北东部城市合作，加快建设通丹经济带，打造近海门户城市、沿边沿线开放城市。继续抓好高速公路、快速铁路、机场等通道工程，建设连接腹地、进关出海通道和东北东部区域交通枢纽。加强医药高新区等开放平台建设，推动各类开发区转型升级。开展务实招商，扩大对外贸易，引进内资年均增长20%，实际利用外资和外贸进出口总额年均分别增长10%。

　　（六）促进文化大发展大繁荣。坚持文化事业和文化产业两手抓、政府扶持和体制改革两加强，促进文化事业和文化产业共同发展繁荣，建设长白山地域文化中心。加强公共文化服务体系建设，文化馆、图书馆、档案馆、体育馆覆盖县（市），公共文化活动场所覆盖乡镇、街道社区和建制村。深入实施文化惠民工程，推动文化企业做大做强，支持中小型文化企业加快发展。建设文化产业园区，打造“国药”、“国砚”、“国参”、“国酒”和剪纸刻纸、广播影视、文化旅游、休闲娱乐等文化品牌，增加相关产业文化含量，文化产业增加值占GDP的比重明显提高。加大文化建设投入，建立文化投资创业基金。加强文化产品创作和专业文艺团体建设，培育造就一批文化名品名人名师和名家。加强文化遗产保护，推动世界文化遗产地、国家重点文物保护单位、历史文化名城保护建设。加强非物质文化遗产挖掘、保护和传承，提升通化文化底蕴和魅力。

　　（七）加强生态文明建设。推行资源节约、环境友好的生产方式和消费方式，精心保护好通化的蓝天绿地、碧水青山和生态资源，建设秀美通化。推动绿色发展、循环发展、低碳发展，抓好污染防治和生态修复，加强水源、湿地、土地、森林、矿产、野生动植物资源保护，建成一批节能减排重点工程，推广沼气等清洁能源，争取进入资源型城市可持续发展试点。严格依法节约集约用地，确保土地高效有序利用。继续开展城乡环境综合整治，基本完成市区周山沟河治理和农村环境连片治理。万元GDP能耗下降12%，化学需氧量、二氧化硫排放量分别下降10%和5%，森林覆盖率达到65%，城镇集中饮用水源地水质达标率100%、生活垃圾无害化处理率达到80%、污水处理率达到70%，重点建制镇全部建成污水处理厂。

　　（八）抓好以改善民生为重点的社会建设。提高群众工资性、经营性、财产性和转移性收入，建立企事业单位和机关工作人员收入正常增长机制。推行更加积极的就业政策，健全覆盖城乡的社会保障体系。推进“八路安居”工程，加快保障性住房建设，完成城市棚户区和农村危房改造。加强社会救助体系建设，保障困难群众基本生活。优化教育结构，合理配置教育资源，大力发展学前教育，均衡发展义务教育，特色发展高中教育，积极发展职业教育，重视发展高等教育，提高教育教学质量。支持通化师院加快发展，规划建设通化大学。抓好人才队伍建设，培养和引进一批技能型、创业型、创新型人才。加强基本医疗保障制度建设，促进基本公共卫生服务均等化，提高医疗救治水平和传染病防控能力。发展体育事业，规划建设全民健身中心。做好人口与计生工作，发展妇女儿童事业，加强未成年人保护，推动残疾人事业和养老服务健康发展。贯彻落实党的民族政策，加大对少数民族事业的扶持力度。抓好双拥共建和军民融合发展，争创全国双拥模范城“八连冠”。改进提供公共服务方式，加强基层社会管理和服务体系建设，增强城乡社区服务功能。落实信访工作责任制，强化安全生产监督管理，推进社会治安防控体系建设，保持社会和谐稳定。

　　三、2013年工作安排

　　2013年，市政府要认真落实市委六届二次全会精神，牢牢把握稳中求进的总基调，全力保持经济持续健康发展，确保各项工作开好局、起好步。预期地区生产总值增长15%以上、突破1000亿元，财政收入增长高于GDP增长，固定资产投资增长30%以上、突破1000亿元，社会消费品零售总额增长14%以上，城乡居民人均收入增长高于全省平均水平。

　　（一）集中力量抓投入上项目。新建续建3000万元以上项目1165项、亿元以上项目303项、10亿元以上项目30项。突出抓好医药工业、生物科技、长白山药谷、兴钢新区、机械制造、镁合金、保健品、循环经济、文化创意、健康养生等10个重点特色园区建设，开工建设桦树医药产业园、中钢汽车新材料产业园，推动通钢新2号高炉、120吨转炉、高强度机械制造用钢竣工投产，加快四长医药工业园、四环制药生产基地、步长北方基地、双龙硅化工产业园等项目实施。加强项目管理和服务，千方百计落实建设条件，促进项目早开工、快建设。对本地企业新上项目给予外引项目同等政策。加强与金融机构合作，发挥各类融资平台作用，提高融资能力。加大招商引资力度，引进亿元以上项目320项，引进资金840亿元，实际利用外资3.2亿美元。完善特色园区和通丹经济带功能定位，推进跨区域合作，建立多层次、宽领域的项目招商和经贸交流机制。启动建设集安边境经济合作区。争取通化全域纳入国家长白山生态保护与经济转型规划。

　　（二）大力提升工业发展水平。深入实施八大工业产业跃升计划，加强银企对接、要素保障和生产运行组织，推动现有企业达产达效，确保工业平稳较快增长。规模以上工业总产值增长15%。抓好医药产业资源整合，尽快形成以研发创新为龙头、以园区化和产业化为主体、以物流服务为配套的发展模式。完成25户医药企业新版GMP认证，医药高新区力争晋升国家级高新区。进一步加大对通钢的支持和服务力度，积极建设“五鸭铁”兴钢新区，争取实现冶金产业恢复性增长。扶持壮大食品产业和骨干企业，开发优质农特产品。提质改造通葡股份，加快通化葡萄酒品牌振兴。开工建设汇源山葡萄酒庄。大力发展镁合金产业，逐步拉长上下游产业链。争取国家政策支持，筹划城区工业企业搬迁改造。抓好能源、建材和保健品、特色生态资源产业发展。加强地质勘探，推进矿产资源节约综合利用。技改投资增长25%以上，新产品产值率超过12%，新创省级以上名牌产品15个。严格医药、食品、机械制造等行业质量监管，扩大国检中心检验检测范围，实现药物生产抽验检验全覆盖。扩大钢铁、医药、食品等工业制成品出口，完成外贸进出口总额6.4亿美元。完成省下达的节能减排指标任务。

　　（三）加强城市建设与管理。严格执行城市规划，完善各专项规划和控详规。城市规划展览馆建成开馆。市区城建计划投资43亿元。加快棚户区改造，拆迁82万平方米，开工建筑面积134万平方米。继续实施“暖房子”工程，完成周边改造及收尾工程77万平方米，对“暖房子”工程进行后续维护管理。全线贯通滨江东路，新建滨江西路江北段堤路工程，升级改造二道江钢城路、东山路，建成江南新区和金厂镇12条路网。开工建设江南新桥，对4座浑江主桥进行设施升级和景观改造，翻建城区3座危桥。实施31条主次街路绿化美化改造提升，推进滨江沿河、街路巷路亮化升级和照明设施改造。实施老城区三期供水管网及附属设施改造工程，抓好城市给水环网改造。新建二道江区净水厂。新增煤气用户5000户，做好天然气置换改造工程前期准备。调增江南、二道江等区域的公交线路和车辆频次。启动周山治理工程，逐步推进南山棚户区改造和银厂区域环境治理。拆迁部分小区遗留平房和棚厦。规划建设停车场。坚持建管并重，实行城市平面、立面及附属设施一体化建设和管理。严格楼盘预售审批和建筑质量监管。进一步加强物业管理。推进“四城”联创，深入开展城市环境综合整治和城乡结合部治理，建设整洁优美的市容环境。

　　（四）促进县域突破和农业农村发展。加快新型工业化步伐，县域工业投资增速超过30%。高标准建设5个县城，推进城乡双向一体化试点，加快节点城镇和特色镇、中心镇建设。开展幸福乡村建设工程，实现“一村一品”特色产业大突破、农村基本公共服务水平大提升、农村综合环境大改变。搞好农业增产增效示范区建设，重大粮食增产技术推广面积达到300万亩以上。抓好20项千万元以上产业化项目，特色农牧产业基地、黑牛养殖加工等项目建成投产。新建130个标准化牧业小区，畜牧业产值增长20%。推进园艺特产业提升，抓好鹿、蜂、蛙、蓝莓等农特产品精深加工。大力发展蔬菜生产，棚膜蔬菜生产基地达到25个，新建蔬菜棚室4000亩，提高冬春蔬菜质量和自给能力。建成长白山人参贸易区和东宝人参产业园，积极开发药食同源新产品，促进人参产业振兴。加强动植物疫病防控，推进农业标准化生产，支持农产品冷链物流发展。启动国家级有机食品基地规划建设。实施第二个十年绿美规划和“三北”防护林五期规划，植树造林10万亩，标准化绿化村屯200个，推动10个林业产业园区加快发展。深入实施“百村示范、全面提升”和“清洁文明提升”工程，力争省级示范村达到120个。抓好农村环境连片整治。发展休闲农业和乡村旅游，筹建10个魅力乡村、100个环城度假村。

　　（五）加快建设基础设施重点工程。继续抓好通梅高速公路建设，官道岭互通立交桥建成通车。做好通靖高速公路开工准备和通集高速公路前期工作，争取尽早开工。通丹铁路进入试运行，力争实现客货通车。积极推进四平至通化至松江河快速铁路前期工作。通化机场启用通航。规划建设通化至三源浦城港经济带。实施通化大街建设工程，对通快一级路进行拓宽改造，升级改造沿线景观和功能设施，建成连接市区和通化县城区的发展带、景观带。统筹推进城乡电网建设，新建扩建66千伏项目10项、10千伏项目11项。启动建设集安界河电站。实施江河堤防整治、水库除险加固等70项水利工程。开工垃圾焚烧发电工程，二道江污水处理厂投入使用。认真落实哈泥河饮用水源环境保护规划，实施人工湿地污水处理工程，抓好一级保护区移民工作和围栏建设。启动二道江矿山地质环境综合整治，完成江南砬头地质灾害治理和绿化彩化。

　　（六）推动改革创新和服务业提速升级。抓好企业重组和产业链整合，稳步推行厂办大集体企业改革。开展中小企业转型升级服务，扶持小微型企业发展，力争成长型企业达到100户以上、“三上”企业突破1000户大关。深化产权制度、土地流转、户籍制度等农村改革。抓好事业单位分类改革。完成市级财政国库集中收付管理制度改革，推行公务卡结算制度，完善公共财政管理体系。抓好企业上市和融资服务，争取5户企业在上海股权托管交易中心挂牌融资。支持农村信用社改制为农村商业银行。规范发展小额贷款公司。加快建设市区、通化县、梅河口物流园区和医药、钢材、农资、粮油等物流项目，封关运营通丹陆港。建成市区蔬菜综合批发市场。建设湾湾川生态新城，带动通快同城化发展。抓好白鸡峰国际养生度假区、龙·溪谷滑雪小镇和辉南旅游示范基地等45个重点旅游项目，加强区域旅游合作，力争开通通丹旅游专列，加强高端媒体宣传，启动高句丽景区5A级创建工作，旅游收入增长34%。

　　（七）着力保障改善民生。制定实施第四个民生工作计划，继续办好百件左右民生实事。开发和稳定就业岗位，增加创业培训补贴，以创业带动就业。城镇新增就业5.5万人，农村劳动力转移就业30万人。增发创业小额贷款，新增个体工商户9000户。提高“五险一金”覆盖率，提高退休人员养老金标准，提高城镇职工和居民医疗保险待遇水平，提高新型农村合作医疗筹资标准，实现城乡养老保险全覆盖。认真解决厂办大集体和五七家属工接续养老保险问题。完善最低生活保障制度，提高低保标准和救助水平。扩大保障性住房受益面，促进商品房市场健康发展。全市完成保障性安居工程45538户，建成廉租房12.6万平方米。市区建设公租房5万平方米，为1.8万户低收入家庭发放住房租赁补贴，完成2800户无籍房确权工作，整治散旧弃管住宅区。改造农村危房1.5万户。建成农村饮水安全工程210处，解决10.1万农村人口饮水安全问题。

　　（八）协调发展各项社会事业。申报国家和省科技项目60项，筹建医药产业技术创新战略联盟，建立医药高新区院士工作站，争取进入全省创新型城市试点和国家知识产权示范市行列。扩大优质学前教育资源，抓好乡镇中小学标准化建设、农村薄弱校改造和特殊教育发展，完成市十三中迁址新建、一中改扩建等工程。实施素质教育，提高基础教育教学质量。推动职业教育与重点企业合作办学，推行劳动者培训、鉴定、就业（创业）一体化模式，加强紧缺性和应用型、复合型人才培养。深化医药卫生体制改革，完善城乡医疗服务体系和公共卫生服务体系。完成市中心医院外科大楼和儿童医院建设工程。继续实施文化强基惠民、地域历史文化资源建设、名人名师名家名品培育和文化产业发展提速工程。建成开放市科技文化活动中心，实现市县乡村公共文化设施全覆盖。规划建设考古遗址公园等文化遗产保护和展示工程，做好全国重点文物保护单位申报工作。成立非物质文化遗产研究院和传习所。加快松花砚产业园区建设，建成吉林省松花砚（石）鉴赏检测中心和网上交易平台，争取关东文化产品交易市场列入国家级版权示范园区。广泛开展文化活动，繁荣文艺创作，打造艺术精品，丰富群众文化生活。大力开展全民健身运动，积极备战十七届省运会和十三届全国冬运会。改造建设市体育场。做好人口和计划生育工作，全面推进免费孕前优生健康检查和幸福家庭创建活动。发展老龄和残疾人事业。筹建东昌区公办社会福利服务中心，加快建设区域性养老中心和社区老年人日间照料站。抓好残疾人托养服务中心建设。推动民族团结进步和民族村镇加快发展。切实加强宗教工作。保护妇女和未成年人权益。深化全民国防教育，巩固和增进军政军民团结。

　　（九）加强和创新社会管理。创新社区管理运行机制，理顺社区岗位职责，加强物业管理等服务。实施重大事项社会稳定风险评估，构建多元社会矛盾纠纷排查调处体系。继续抓好“六五”普法。认真做好信访工作，切实解决群众合理诉求。强化安全生产工作领导，加大安全生产投入，努力创建本质安全型城市，遏制重特大安全事故发生。加强社会治安综合治理，深入推进治安防控体系建设，积极预防和惩治违法犯罪。完善监管体系，建立健全综合协调机制，严厉打击制售假冒伪劣食品药品犯罪，保障群众饮食用药安全。加强物价监管，稳定生活必需品市场价格。加强应急管理，有效预防、妥善处置突发事件。搞好气象、地震等防灾减灾工作。深入实施“强基富民固边”工程，维护边境稳定和国家安全。

　　（十）进一步加强政府自身建设。完成历史使命，实现宏伟目标，要求我们全面提高政府的公信力、执行力和推动力，努力建设法治政府、效能政府、服务政府、廉洁政府。坚持依法行政，规范行政行为，严格、公正、文明执法。自觉接受人大及其常委会的法律监督、工作监督，主动接受政协的民主监督，认真听取民主党派、工商联、无党派人士和各人民团体的意见，高度重视司法监督、舆论监督和社会公众监督。落实决策听证、实施效果评估等制度，科学决策、民主决策、依法决策。深化行政审批制度改革，简化审批流程，提高审批效率。深入推进政务公开，保障人民群众的知情权、参与权、监督权。加快工作节奏，做实工作措施，细化工作组织，创新工作思路，狠抓工作落实，敢于开拓，勇于担当，少说多做，说到做到。关注基层，关心百姓，千方百计为企业和群众排忧解难。切实改进会风文风，从文山会海中解脱出来，把精力放在调查研究、谋划发展和推动工作上。完善绩效管理，严格行政问责，提高行政效能，实现办事提速、管理提效、服务提质。加强软环境建设，深入开展群众评议活动，整治庸懒散奢，克服形式主义、官僚主义，严肃查处涉软案件。倡导艰苦奋斗，勤俭办一切事情，降低行政成本，建设节约型机关。全面推进惩治和预防腐败体系建设，加强行政监察、审计监督，塑造干部清正、政府清廉、政治清明的良好形象。全体公务员特别是领导干部要铭记人民重托，保持昂扬向上的精神状态、干事创业的工作激情，勤勉尽责，廉洁自律，争先创优，做人民满意的公仆。

　　各位代表！我们已经踏上了全面建成小康社会新的征程。让我们深入学习贯彻党的十八大精神，在市委的坚强领导下，在市人大、市政协的大力支持下，解放思想，开拓创新，扎实工作，为推动通化科学发展、加快振兴，让全市人民生活得更加美好而努力奋斗！
